# “Migratory beekeeping and its influence on the prevalence and dispersal of pathogens to managed and wild bees”

**DOI:** 10.1016/j.ijppaw.2022.05.004

**Published:** 2022-05-21

**Authors:** Vicente Martínez-López, Carlos Ruiz, Pilar De la Rúa

**Affiliations:** a Department of Evolution, Ecology and Behaviour. Institute of Infection, Veterinary and Ecological Sciences. University of Liverpool. Liverpool, L69 7ZB, UK; b Department of Animal Biology, Edaphology and Geology, Faculty of Sciences, University of La Laguna, La Laguna, Tenerife, Spain; c Department of Zoology and Physical Anthropology, Faculty of Veterinary, University of Murcia, 30100, Murcia, Spain

**Keywords:** Migratory beekeeping, Stationary beekeeping, Pathogens, Parasites, Colony losses, Genetic impact

## Abstract

Demand for food is growing along with the human population, leading to an increase in plant production. Many crops are pollinated by insects, so the global demand for managed pollinators is also increasing. The honey bee has traditionally been considered the main provider of crop pollination services. For providing it beekeepers seasonally transport hives to different locations after the flowering of different crops. These movements could be detrimental to pollinators by: i) stressing honey bees, making them more susceptible to pathogens and parasites; ii) spreading bee parasites and pathogens across locations; iii) increasing the transmission of parasites and pathogens between managed and wild pollinators and vice versa (spillover and spillback, respectively). To understand the impact of migratory beekeeping on bee health, we conducted a systematic review to identify the main trends and provide a complete picture of existing knowledge on the subject. We found 52 studies analysing pathogen-related impacts of migratory beekeeping on honey bees. However, only 16 investigations tested the effect of migratory practices on the prevalence and spread of pathogens and parasites. We found no studies that assessed the impact of migratory beekeeping on the occurrence and spread of pests and diseases in wild bees. In general, migratory beekeeping tends to increase the prevalence of pathogens and parasites in honey bee colonies. However, the results were very heterogeneous, probably due to several uncontrolled underlying factors such as management, biological and geographical factors, and the interactions between them. In conclusion, there is an urgent need for studies to assess the impact of migratory beekeeping on bee health, given the current global bee decline and the expected increase in migratory beekeeping due to climate change and crop pollination demand.

## Highlights

• Migratory beekeeping may affect the health of both managed and wild bees.
• Only 16 studies compare the prevalence of pathogens between migratory and stationary hives.
• Results are heterogeneous due to the complex interaction of the underlying factors.
• Overall, the prevalence of pathogens and parasites increased in migratory honey bees.
• The impact of migratory beekeeping on the health of wild bees is unknown.

## Graphical abstract

**Figure:**
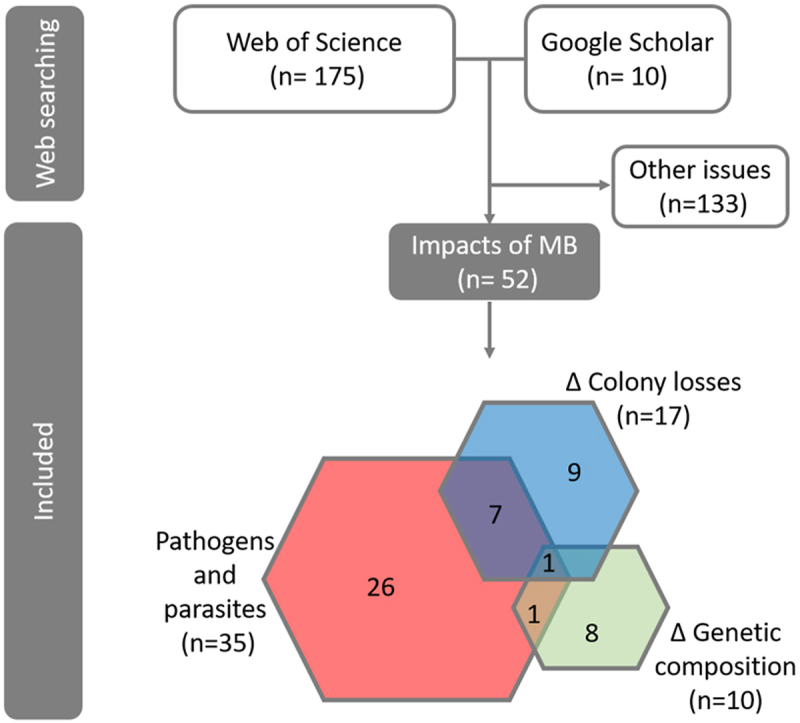

## 1 Introduction

Global demand for food inexorably pushes us towards an increase in plant production directly for human use and indirectly through livestock feed (van Zanten et al., 2016). This growth is characterised by a trend towards intensification of agriculture in the form of monocultures and pollination-dependent crops (Aizen et al., 2019), which has indirectly led to a decline in wild pollinators (Raven and Wagner, 2021) and an increased reliance on managed pollinators to ensure plant production (Osterman et al., 2021). Therefore, demand for crops requiring insect-mediated pollination has risen (Aizen and Harder, 2009). Although the most diverse group of pollinators is the Lepidoptera (140,000 species; Wardhaugh, 2015), Hymenoptera (70,000 species) and especially bees (20,000 species; Michener, 2000) are considered the most important pollinators because their body characteristics often allow them to transport large numbers of pollen grains, and they are generally found in high abundance in both natural and agricultural ecosystems (Ollerton, 2017). Bees pollinate most fruit and vegetable crops that provide essential nutrients, enrich the diets of human populations (Eilers et al., 2011) and mitigate essential nutrient deficiencies (Smith et al., 2015). Therefore, maintaining efficient pollination strategies is increasingly important (Isaacs et al., 2017; Cheptou, 2021).

It is known that wild and domestic bees provide comparable amounts of pollination for most crops, even in intensively cultivated regions (Reilly et al., 2020), yet honey bees are the most dominant species globally both in natural (Hung et al., 2018) and agricultural (Allen-Perkins et al., 2021) ecosystems. Additionally, it is the most frequently reported as the main pollinator of the most important crops as perceived by growers (Garbach and Morgan, 2017). This is reflected in crops such as almonds, for which an entire beekeeping industry has focused on providing large numbers of honey bees in the United States. In fact, during almond blossom, two-thirds of all honey bee colonies in the country are used to pollinate Californian almonds (American Beekeeping Federation, 2018). The estimated value of pollination service revenue from managed honey bee pollination in the United States (USA) is approximately US$17 billion per year (Calderone, 2012). Across the European Union (EU), insect pollination of crops accounted for €14.6 [±3.3] billion annually, equivalent to 12 (±0.8) % of the total economic value of annual crop production (Leonhardt et al., 2013).

In order to meet the demand for pollination, honey bee colonies are transported at different distances that vary greatly depending on the area of the country and the crops visited. In the most extreme cases, such as those occurring in the USA, colonies are transported at continental scale across several states by trucks to a range of monocultures such as blueberries, cranberries, almonds, and citrus (vanEngelsdorp et al., 2013) for months at a time. At each stop on the journey, millions of honey bees from different origins converge on a single crop during flowering, which usually lasts about a month and may offer little forage diversity (Colwell et al., 2017). However, not only in Europe but also in Australia and China, such movements often take place nationally between and within regions to take advantage of different blooms to offer monofloral honeys to the consumer and to allow honey bee colonies to cope with the adverse conditions of increasingly hot and dry summers (Jara et al., 2021).

Over the last 50 years we are witnessing a decline in the species richness of wild bees and other pollinators (Zattara and Aizen, 2021). Factors considered responsible for this decline include habitat loss, with a consequent reduction in the abundance and diversity of floral resources and nesting opportunities, and exposure to cocktails of agrochemicals and to emerging parasites and pathogens that are accidently moved around the world due to human action (Goulson et al., 2015). Recent studies have shown that stress factors do not act in isolation but synergistically (Siviter et al., 2021) and that their interactions can be difficult to predict; for example, both pesticide exposure and food stress can alter the immune response, making bees more susceptible to parasites. While pathogens and diseases are mainly threatening managed European honey bees (Steinhauer et al., 2018), the decrease in wild bees, among other flying insects, seems to be especially driven by land-use change and agricultural intensification (Hallmann et al., 2017; Raven and Wagner, 2021). In any case, recent studies have demonstrated the transmission of pathogens from honey bees to wild bees and vice versa (known as pathogen spillover and spillback, respectively) (Martinez-López et al., 2021; Nanetti et al., 2021; Piot et al., 2022). This spillover is partly mediated by the presence of infected honey bees in different habitats (González-Varo et al., 2017; Manley et al., 2019), which can deposit pathogens on flowers and thus cause them to act as vectors for pathogen transmission between different pollinator species (Singh et al., 2010; Graystock et al., 2015, 2020).

Given the increased trade in managed honey bees for pollination purposes in recent decades, the transmission of pathogens has been fostered. This widespread transmission can occur at the landscape scale following the release of managed hives into a new settlement, and also across a region if managed pollinators are used on a large scale, such as during honey bee hives migratory events. As the beekeeping sector has a direct influence on ecology, crops, environment and biodiversity, it is necessary to develop research and conservation strategies for these managed pollinators, with the aim of ensuring the sustainable growth and maintenance of the honey bee populations, its health and genetic diversity, and the improvement or modernisation of management techniques. To this end, it is necessary to promote the identification of criteria for the adequacy of beekeeping stocking rates that make the sustainable use of beekeeping resources compatible with the conservation of wild bees. In this article we aim to conduct a systematic review of studies published to date focusing on: i) the impact of migratory beekeeping on the health of honey bees; and ii) the unintentional spread of pathogens to and from managed honey bees to wild bee populations due to the movement of hives that may contain diseased colonies.

## 2 Material and methods

### 2.1 Search strategy and records selection

A systematic literature review was conducted in the Web of Science (https://www.webofscience.com/) using the terms: “(migratory beekeeping OR mobile beekeeping OR nomadic beekeeping OR transhumance beekeeping)” together with “AND (pathogen)” or “AND (parasite)” for articles published until January 2022. Additionally, we also performed specific bibliographic searches with the names of the most common bee pathogens (e.g. virus, microsporidia, etc). For the topic search, all possible combinations were used. In addition, we performed an active search for articles using Google Scholar (https://scholar.google.com) to ensure that we got a complete picture of all available information.

The Preferred Reporting Items for Systematic Reviews and Meta-Analyses (PRISMA) guidelines (Moher et al., 2009) were used to conduct the systematic review. After listing the records found in each database, duplicates were removed. Next, titles and abstracts were examined and records that did not cover the search keywords were removed. Finally, full texts were screened for eligibility using the following exclusion criteria: i) language other than English; ii) review articles and checklist; iii) topics outside the study question.

### 2.2 Data collection and analysis

Records were carefully reviewed to exclude studies that: only mentioned or described the practice of migratory beekeeping in specific areas; focused on assessing the cost-effectiveness of migratory beekeeping; developed new technological tools to assist beekeepers in making migratory movements more cost-effective (i.e. finding optimal places or routes). Finally, the studies obtained after this filter were examined in detail to extract the relevant information: the hypothesis tested, pathogens/parasites assessed, the effect of migratory beekeeping on pathogens/parasite prevalence and main conclusion(s) of the study.

### 2.3 Number of migratory hives per country

Information on the practice of migratory beekeeping (=honey bee transhumance) in different countries was sought from official government sites, as well as on scientific literature.

## 3 Results

**Fig. 1:**
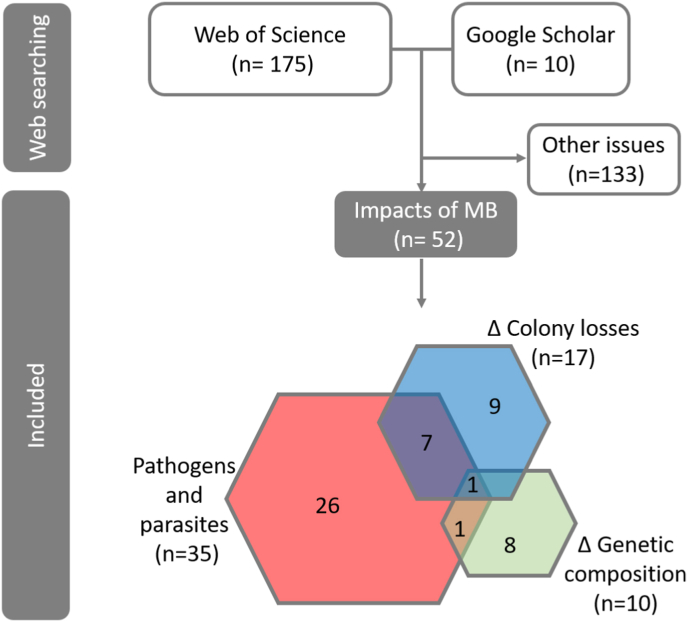
Flow chart of the process of the systematic review on the different impacts of migratory beekeeping (MB), including the number of studies analysed at each step of the review process. Detailed data in Supplementary Table 1.

Following the search process we reviewed 185 articles, 175 from the Web of Science and ten from Google Scholar. After applying the analysis filters, 133 articles were discarded, leaving a database with a total of 52 studies analysing the pathogen-related impacts of migratory beekeeping on honey bees (Fig. 1; Supplemental Table 1). As we have not found any studies on the impact of migratory beekeeping on the prevalence of pathogens and parasites in wild bees, our second objective could not be developed.

Of the 52 articles (Supplemental Table 1), 35 were found to be related to pathogens and parasites associated with migratory beekeeping in honey bees. The remaining studies were related to the effect of hive movement on colony losses (17 studies) and the impact of migratory beekeeping on the integrity of the genetic composition of honey bee subspecies and populations (10 studies). Eight of these studies assessed some of these issues together, but only one study was found in which all these aspects were analysed together. Of the studies related to pathogens and parasites, only 16 of them explicitly assessed the impact of migratory beekeeping on pathogens and parasite prevalence in managed honey bees. The remaining were related to the contribution of migratory beekeeping to the spread of pathogens and parasites or to the prevalence of infectious agents in migratory colonies.

Of the resulting 16 studies that test the effect of the migratory practices on pathogen prevalence and spread, most of them (n = 9) were done in the USA, three in South Africa, two in Spain, one in Turkey and one in Brazil (Table 1). Given the importance of this beekeeping practice in the USA and specifically in California for the pollination of almond trees, more studies have been carried out on this continent than in Europe.

**Table 1:**
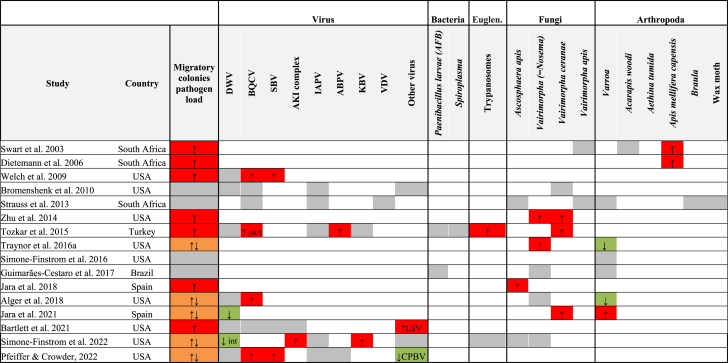
List of studies included in the analysis of the influence of migratory beekeeping on the prevalence of pathogens or parasites. Colours indicate the level of prevalence in migratory colonies compared to stationary colonies: higher (red), lower (green), no significant differences (grey), orange: both increase and decrease, part: only in some colonies, int: interaction between factors.

Among the pathogens analysed in the included studies, there are ten viruses in a total of nine articles, two bacteria genera in two papers, two fungi genera in 11, of which ten correspond to species of the genus *Vairimorpha* (=*Nosema*) (hereafter, *Vairimorpha*; Tokarev et al., 2020), eight on different arthropod species in six of which the spread and prevalence of the *Varroa* mite is discussed, and only two articles analysed the prevalence of trypanosomatids and how this varies with the development of migratory beekeeping.

**Fig. 2:**
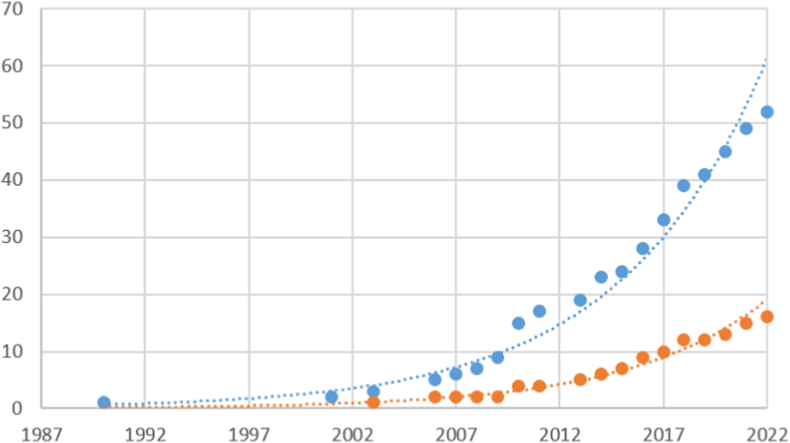
Cumulative number of publications examining in general the impact of migratory beekeeping (blue dots) and in particular the prevalence of pathogens (orange dots) from 1990 to 2022. Exponential trend lines are represented by dashed lines. (For interpretation of the references to colour in this figure legend, the reader is referred to the Web version of this article.)

In terms of the number of publications in the databases used to obtain the database analysed here, there has been an exponential growth in the number of articles related to the impact of migratory beekeeping (Fig. 2). There has also been an exponential increase in the number of articles related more specifically to the effects due to contact between migratory and stationary hives on the prevalence and dispersal of pathogens.

Migratory beekeeping was found to produce several impacts on the honey bee colonies that could be related with pathogen and parasite prevalence.

### 3.1 Effect of migratory beekeeping on the prevalence of pathogens and parasites

#### *3.1.1* Migratory vs stationary beekeeping

Only a limited number of observational surveys (16 studies) compared pathogen prevalence between colonies with both types of management (migratory and stationary hives). Twelve of them found a significantly higher prevalence in migratory colonies in some of the pathogens or parasites studied (Table 1). On the other hand, five of them found that migratory colonies showed a significant decrease in pathogen prevalence of DWV (Jara et al., 2021; Simone-Finstrom et al., 2022), varroa mites (Traynor et al., 2016a; Alger et al., 2018) or CBPV (Pfeiffer and Crowder, 2022).

The first study on the impact of migratory beekeeping on pathogen and parasite prevalence was performed in South Africa. This research reported a higher but not significant prevalence of the microsporidium *Vairimorpha apis* and the tracheal mite (*Acarapis woodi*) in migratory colonies than in stationary ones (Swart, 2003). Furthermore, the author reviewed the information available about the spread of the parasitic Cape honey bee (*Apis mellifera capensis*) into the distribution area of the African honey bee (*Apis mellifera scutellata*) due to migratory operations. Cape honey bee workers can enter African honey bee colonies and lay their own eggs, acting as parasites that eventually lead to hive collapse (Allsopp, 1992). In this regard, Dietemann et al., (2006) found that parasitic infestation of the Cape honey bee on African honey bees was higher in migratory hives than in stationary ones. However, parasitic loads were smaller only in those stationary apiaries isolated from migratory operations. Contrastingly, other studies which analysed the prevalence of a wide range of pathogens and parasites in South Africa did not find any difference in pathogen and parasite prevalence between migratory and stationary colonies (Strauss et al., 2013).

Recent studies have focused on several pathogens and parasites. With regard to honey bee viruses, stationary hives showed in general lower prevalences (Table 1). BQCV was found more prevalent in migratory colonies in the USA (Welch et al., 2009; Alger et al., 2018; Pfeiffer and Crowder, 2022) and Turkey (Tozcar et al., 2015). However, some of these differences were transient as BQCV prevalence were equal in migratory and stationary colonies after one month in some cases (Alger et al., 2018). ABPV had higher prevalence in migratory colonies (Tozcar et al., 2015) while DWV prevalence has been found to be higher in stationary colonies (Jara et al., 2021; Simone-Finstrom et al., 2022) or did not show any significant trend (Welch et al., 2009; Bromenshenk et al., 2010; Tozcar et al., 2015; Alger et al., 2018; Bartlett et al., 2021, Pfeiffer and Crowder, 2022). Multiple viral infections are not uncommon in honey bees (Chen et al., 2006), but seem to increase in migratory beekeeping operations, where triple infections were found at a higher prevalence (Welch et al., 2009).

On the other hand, *Vairimorpha* (= *Nosema*) *ceranae* was reported to have a higher prevalence in migratory colonies (Zhu et al., 2014; Tozcar et al., 2015; Jara et al., 2021). Other studies also reported higher *Vairimorpha* spp. spore loads in migratory colonies (Traynor et al., 2016a), but this trend was not uniform as some studies did not find any difference in *Vairimorpha* spp. prevalence between migratory or stationary colonies (Guimarães-Cestaro et al., 2017; Alger et al., 2018; Simone-Finstrom et al., 2022). The information about *Varroa destructor* was highly heterogeneous with some studies reporting a significant increase in varroa loads in migratory colonies (Jara et al., 2021), a non-significant effect of the management (Strauss et al., 2013; Simone-Finstrom et al., 2016; Guimarães-Cestaro et al., 2017) or a higher prevalence in stationary operations (Traynor et al., 2016a; Alger et al., 2018). Interestingly, Alger et al. (2018) did not find any impact of the movement of hives on varroa load when the migratory colonies had just arrived, but one month later mites load was higher in stationary colonies. The fungi *Ascosphaera apis* was found more frequently in migratory colonies in Spain (Jara et al., 2018) but no trend was found in other studies (Strauss et al., 2013; Simone-Finstrom et al., 2022). Finally, trypanosomes were just assessed twice and in one study they were more prevalent in migratory colonies (Tozcar et al., 2015).

Additionally, there was one study focused on the development of resistance to treatments for *V. destructor* depending on the management of the honey bee hives (migratory *vs* stationary) (Rodríguez-Dehaibes et al., 2011). Interestingly, these authors found an increase of the resistance to treatments in stationary colonies.

#### 3.1.2 Pathogens prevalence in migratory colonies

The prevalence of pathogens and parasites was assessed only in migratory colonies in seven studies conducted in the USA (Supplemental Table 1). Spivak and Reuter (2001) found that migratory beekeeping tends to homogenise the mite loads among the different hives. This happens when colonies from different origins are placed together in a common location, and hence, infected honey bees can drift from one colony to another (e.g. when robbing resources) spreading the mites across hives. In general, most of the studies found that the prevalence of varroa mites were the main factor predicting honey bee health status (Glenny et al., 2017; Drummond et al., 2021). The prevalence of varroa outperformed the role of other pathogens such as *A. woodi*, viruses, trypanosomes and microsporidia compromising the survival of migratory honey bee colonies (Drummond et al., 2021).

Various studies identified the date of sampling as a key factor in the prevalence of the different pathogens and parasites (Runckel et al., 2011; Glenny et al., 2017; Faurot-Daniels et al., 2020). For example, Runckel et al. (2011) found a strong seasonality in pathogens prevalence: some honey bee viruses and *Vairimorpha* spp. peaked in summer while the trypanosomatid *Crithidia mellificae* and the virus LSV-2 were more prevalent in winter. In the same line, Glenny et al. (2017) and Faurot-Daniels et al. (2020) detected that, in general, pathogen prevalence increases from winter to summer, although contrasting patterns were found in some cases (e.g. DWV increased from summer to fall while LSV-2 decreased in the same period, Faurot-Daniels et al., 2020).

The exposure of honey bees to agricultural landscapes seemed not to have an impact on varroa or *Vairimorpha* prevalence in spite of a higher exposure to pesticides (Meikle et al., 2017). Furthermore, queen events and generally unknown illnesses such as the Idiopathic brood disease increased the risk of colony collapse in migratory beekeeping operations (vanEngelsdorp et al., 2013).

### 3.2 Impact of migratory beekeeping on colony losses and genetic diversity

There were 17 studies that related winter colony loss rate with beekeeping practices, but they showed mixed effects. Most of the studies (35%) found an increase in colony losses due to migratory beekeeping, even doubling the rate of hive losses (Pirk et al., 2014). These results were found in several continents such as North America (Bromenshenk et al. 2010), Europe (Gray et al., 2020), Asia (Tozkar et al. 2015) or Africa (Dietemann et al., 2006; Pirk et al., 2014). However, some studies found no significant differences in colony losses between migratory and stationary hives (30%) or even a decrease of these events (35%) in migratory operations. In countries such as the USA (vanEngelsdorp et al., 2010) or Austria (Oberreiter and Brodschneider, 2020) a lower rate of colony losses in winter was found in migratory compared to non-migratory beekeeping operations. The reasons evoked to explain these results were that beekeepers with migratory operations have more experience in beekeeping practice and that migratory colonies have access to better foraging sources. Eight of these studies that analysed the prevalence of pathogens and colony loss in a joint analysis, resulting in contradictory results with migratory colonies showing either an increase in pathogens (i.e. *V. ceranae*: Tozkar et al., 2015; Jara et al., 2021, ABPV: Tozkar et al., 2015; LSV: Bartlett et al., 2021; KBV and AKIV: Simone-Finstrom et al., 2022) or parasites (i.e. *A. m. capensis*: Dietemann et al., 2006) prevalence or on the contrary a decrease in some pathogens loads (i.e. DWV: Jara et al., 2021; Simone-Finstrom et al., 2022).

The impact of migratory beekeeping on genetic diversity was analysed in 10 studies and showed more concordant results among the different studies, with a large majority (80%) showing genetic change in the migratory colonies. Only two studies in Spain found no significant genetic differences between both types of management (Arias, 2006; Jara et al., 2021).

### 3.3 Migratory beekeeping practice in different countries

In general in the EU there is a certain disparity when it comes to obtaining information on the practice of migratory beekeeping: while in countries such as Denmark (Kryger pers. comm.), Greece (Bouga pers. comm.) or Portugal (Pinto pers. comm.), there is no official information on the number of hives moved (although there is evidence that it is carried out), in others such as Spain, Romania and Italy it is more detailed.

Spain presents the highest number of hives in the EU and also the highest level of professionalisation with 18% of professional beekeepers (those who managed more than 150 hives per apiary). These professional beekeepers managed most of the existing hives: of the three million hives in Spain, 80% belong to professional beekeepers (MAPA, 2020). The number of migratory apiaries (around 14,000 in 2021) has doubled in the last 10 years compared with the non-migratory ones that have remained constant, which may be partly due to the subsidy beekeepers receive for this beekeeping practice. Migratory beekeeping movements take place mainly in the centre and south/southeast of the Iberian Peninsula, but there are regions such as the Canary Islands where a sharp change in the type of management has been observed in recent years with an increase of migratory colonies (from 22% of the total number of hives in 2014 to 51% in 2020) (ISTAC, 2020). In Romania, the second country in the EU in terms of the number of hives (2,247,000), 27.9% of them are included in migratory movements and 30% in mixed beekeeping practices (Pocol et al., 2021). In Italy (Mutinelli, pers. comm.) the percentage of apiaries carrying out migratory beekeeping is 44%. This value is higher than 80% in southern regions such as Calabria (85%) and Sicily (83%) but it is also a very frequent practice in northern regions such as Valle D'Aosta (84%) and Piedmont (74%). In Austria colonies migrate mainly to harvest special honeys, while migratory beekeeping for a paid pollinator service is rarely used (Oberreiter and Brodschneider, 2020), but again there are no statistics on the number of moving apiaries. Migratory beekeeping has been practised in the Carpathian Basin for at least 300 years (Pinke et al., 2021) to produce monofloral honeys and has been shaped by changes in cropping patterns. Within the EU, migratory beekeeping can take place between neighbouring countries such as Germany and Denmark where approximately 400 hives are moved each year, both for the pollination service and for the health benefits of consuming clover pollen. In western Denmark, there are landscapes with a lot of heather, *Calluna vulgaris*, and many beekeepers move their hives to these areas for the attractive honey (Kryger, pers. comm.).

In the USA, there are an estimated 2.7 million commercial honey bee colonies (i.e. those that are migratory), more than half of which are contracted to pollinate a variety of crops (USDA, 2021). To meet this demand, large numbers of honey bee hives are moved between crops regionally and nationally (Alger et al., 2018). The conditions of migrating hives vary greatly depending on the distance travelled and the crops visited. In the most extreme cases, colonies are trucked to a range of monocultures including blueberries, cranberries, almonds, and citrus (vanEngelsdorp et al., 2013) for months at a time. At each stop on the journey, millions of honey bees from different origins converge on a single crop during flowering, which usually lasts about a month and may offer little forage diversity (Decourtye et al., 2010; Colwell et al., 2017). The crop with the greatest pollination needs is primarily California almond, which required approximately 81% of USA honey bee colonies in 2018 (USDA, 2018). In that year over 1.8 million hives were sent to California, a figure three times the number of hives native to California (USDA, 2018). This demand for almond pollination constitutes approximately one-third of USA beekeeping revenue, and many commercial beekeeping operations depend on the almond pollination market (Lee et al., 2019). Other migratory movements of honey bee hives in the USA involve the pollination of wild berries, such as blueberries. In this regard, more than 80,000 hives were brought to Maine in 2016 to pollinate wild blueberries (Lund, unpublished data in Drummond et al., 2021).

In Saudi Arabia, most beekeepers practice hive migration to avoid extreme weather conditions or lack of food. A survey found that 93% of beekeepers reported moving their hives 2–9 times a year (Adgaba et al., 2014). The high domestic demand for honey and the high price it fetches make it a very profitable activity. However, most beekeepers (71%) use traditional hives instead of modern hives. Several authors (Ansari et al., 2017, Poole, 2021) have concluded that the importation of honey bees, together with the high mobility of hives, poses a major threat to the spread of honey bee diseases in the country.

In India, migratory beekeeping with *Apis mellifera* allows 4–5 harvests per year with an average annual yield of approximately 50–60 kg per hive, which represents a significant economic income (Sharma et al., 2013, Kishan Tej et al., 2017). In addition, migration improves the strength of the colonies (Brar et al., 2018). Migratory beekeeping is practised both within states and on a large scale between states. In northern India, transhumance occurs between plains and hilly areas, taking advantage of flowering crops such as rapeseed, mustard, lychee orchards or sunflower fields. In southern India, they take advantage of the flowering of rubber and tamarind plantations (Kishan Tej et al., 2017).

In China, where there are approximately six million *A. mellifera* hives (CNCAGR 2011), the demand for honey bees for pollination in agriculture is receiving increasing attention (Zheng et al., 2018). Honey bee colonies rented or purchased for pollination of watermelons and strawberries in greenhouses are mainly from sedentary apiaries, located close to farms or greenhouses. Although it is estimated that 80% of *A. mellifera* beekeepers migrate across the country in search of different honey streams (Zheng et al., 2018), the pollination market is not developed enough to provide sufficient business for migratory apiaries. Basically, colonies move from southern China to the north and then from the north to the south every year. These long-distance transhumance movements can be over 3,000 km each year although there are shorter ones. On the other hand, of the five endemic species in China, the use of *A. cerana* in pollination is also increasing (Zheng et al., 2018), which has led to a high demand for colonies of this species.

In Australia there are about 600,000 hives (RIRD, 2007) and in states such as New South Wales about 200,000 are managed by commercial beekeepers (those with more than 201 hives). Renting hives to farmers who benefit from honey bee pollination is an important source of income. This paid pollination takes place in most states and is most important in the almond orchards of Victoria and South Australia. It is estimated that 100,000 hives will be required for almond pollination over the next two years. Despite large equipment and subsidies for their purchase, most beekeepers only move their hives as often and as far as necessary because they prefer to work in their localities. Typical long journeys are to the aforementioned almond pollination, and, in case of drought, to any place where it has rained. On the other hand, as stated by Clemson (1985) in RIRD (2007) “virtually all commercial honey production in Australia is from hives that are moved (migrated) from one source of pollen and nectar to another”.

## 4 Discussion

### 4.1 Migratory beekeeping definition

In order to design effective management measures to mitigate the effects of pathogens spread on bees, a first step is to define the concept of migratory beekeeping (also called nomadic or mobile beekeeping or transhumance of honey bee hives). This is not so simple because in each territory the purpose and dimension of this beekeeping practice changes, so it has to be adapted according to the geographical area where the assessment is carried out. An initial definition to elaborate on is that migratory beekeeping is the seasonal transport of hives by beekeepers. The reasons for moving hives have been diverse. Traditionally, it has been done to maximise honey production according to the availability of floral resources due to differences in flowering time at different altitudes or latitudes. This ancestral practice is widespread in the Mediterranean basin and there are numerous written records from antiquity on the transport of beehives by land (with animals) and even by sea or rivers (with ships) around the Mediterranean area (Pliny 21.73–78, Columella, Rust. 9.14.19) to exploit floral resources. However, in recent decades, migratory beekeeping has been promoted as a pollination service for economically important crops such as almond or apple orchards. The movement of honey bee colonies to service pollination contracts with agricultural producers or to support honey production for profit is also known as commercial beekeeping in countries such as the USA or Australia where the vast majority of hives are migratory. However, in many cases trade in honey bee products does not involve transhumance movements to obtain them. Therefore, two types of migratory beekeeping could be defined: for honey production and for providing the service of pollination. Both types of hive movement involve different management, with potentially different implications for pathogen and parasite spread. For instance, hives involved in pollination services might have higher exposure to pesticides (e.g. Traynor et al., 2016b) and worse nutrition in the case of intensified agricultural landscapes with monocultures and/or crops with poor quality pollen or low nectar production (Colwell et al., 2017). However, hive locations for honey production are selected based on the quality of floral resources which generally means better nutrition. We thus recommend that future studies take these differences into account when assessing the impact of migratory beekeeping on honey bee health.

### 4.2 Impact of migratory beekeeping on pathogen and parasite prevalence

Migratory beekeeping and managed honey bee trading have allowed the spread of pathogens and parasites such as *Vairimorpha ceranae*, *Varroa destructor* and *Ascosphaera apis* among others (Owen, 2017) globally, regionally and locally. It is well-documented that migratory beekeeping had an important role in the propagation of *A. woodi* in Mexico (Eischen et al., 1990). More recently, migratory beekeeping has been crucial for the spread of the small hive beetle (*Aethina tumida*) across countries within continents, and for this reason, a ban on migratory beekeeping in areas where the parasite is spreading has been proposed as an essential measure to control its dispersal (Schäfer et al., 2019). Therefore, migratory beekeeping should implement measures to avoid the spread of honey bee pests and diseases. In that sense, molecular tools can help to perform fast, cheap and extensive testing programmes to detect the presence of honey bee pathogens and parasites (Grangier et al., 2015; de Miranda et al., 2021). On the other hand, it is also important to keep national records of hive movements performed by beekeepers, a procedure already performed in some countries (e.g. Spain). This should help to unravel the pathways of pathogens and parasites spread by identifying locations that are connected by migratory movements. For instance, Gordon et al. (2014) analysed how migratory beekeeping movements in south-eastern Australia connected different locations in the area by applying a network approach.

There were a limited number of studies testing the effects on migratory and stationary colonies, and most of them found higher prevalence of pathogens or parasites in migratory ones. This could be explained by several reasons such as i) higher oxidative and heat stress (Simone-Finstrom et al., 2016; Melicher et al., 2019), ii) higher probability to come into contact with other colonies, which increases the risk of pathogen transmission (Welch et al., 2009), iii) higher exposure to pesticides (Traynor et al., 2016b), iv) increased brood rearing (Jara et al., 2021), and v) a reduction in forage diversity in pollination service operations (Colwell et al., 2017). However, studies showed highly heterogeneous results on the impact of migratory beekeeping on the prevalence and spread of pathogens and parasites, probably due to several uncontrolled underlying factors that may affect the outcomes of the performed studies, such as management, biological and geographical factors, and the interactions among them.

An important factor to consider in studies is the **management** of the hives by the beekeepers, that is known to influence the pathogen prevalence (e.g. Jara et al., 2021; Bartlett et al., 2021). As a general rule, the management of large-scale migratory beekeepers is very different from the techniques used by small-scale, mainly stationary beekeepers (Welch et al., 2009; Bartlett et al., 2021), which is generally not reported or under-described in most studies. Some key aspects such as preventative and curative pest controls, operation size, supplemental feeding, queen replacement or the age of the queens, can affect the results obtained. Additionally, there are large differences in the migratory beekeeping practices (operation size, treatments, migratory purpose, …) among regions and countries making it difficult to interpret the observed differences between studies. The migratory beekeeping practised in the USA, where hives are transported at continental scale for thousands of kilometres, differs greatly from the generally short movements performed in Europe, Africa, or South America (see Table 1). Greater distances between the origin and the final destiny of hives would potentially mean a higher oxidative and heat stress of the honey bees by increasing the time of transportation (Simone-Finstrom et al., 2016; Melicher et al., 2019). Furthermore, there are marked differences in management. For instance, there are differences in the chemical products authorised to treat the pathogens and parasites that affect honey bees: the use of some products to treat *V. ceranae* infection is allowed in the USA but not in the EU which might complicate obtaining conclusive results on the impact of migratory beekeeping on pathogen prevalence. For example, there were opposing results in Varroa mite prevalence between migratory and stationary beekeeping operations in two recent studies (Alger et al., 2018; Jara et al., 2021). These differences may be due to the differential management between the two studies, in terms of the distance travelled (4,300 *vs* 430 km), the purpose of the migration (almond pollination service *vs* honey production), supplemental feeding (present *vs* absent), mite treatment as well as differences in colony size. This mite is known to be the main vector of several viruses, affecting the load of several viruses (Le Conte et al., 2010). Thereby, investigations assessing the impact of migratory beekeeping on honey bee health should always frame their conclusions taking into account the peculiarities of the beekeeping in their study area to avoid the emergence of apparent contrasting results that can not be actually compared.

There are several **biological factors** that should be considered for explaining differences in pathogens prevalence. For example, several recent studies have found that the effects of migratory practices on pathogen level varied with age, behavioural and life-history stage (Simone-Finstrom et al., 2022). Adult honey bees had higher loads of rare viruses such as AKI complex followed by higher levels of DWV and Varroa in the late season. Also, they found an overall decline of IAPV and KBV viral loads and EFB in foragers compared to in-hive honey bees. Additional biological factors, such as the richness of bee species present in each area, the relative abundance of social bee species or the abundance of floral resources, drive the temporal dynamics of parasites (Graystock et al., 2020). Furthermore, there is evidence that floral traits affect pathogen transmission (Adler et al., 2021), suggesting that it will also be modulated by flower availability and species diet breadth (Proesmans et al., 2021).

It is also important to take into account several **geographical factors** that could explain these heterogeneous results such as i) landscape composition, ii) differential agrochemical exposure or iii) climatic effects. Landscape composition can be an important element in understanding potential inter- and intraspecific pathogen transmission by concentrating or diluting pollinator interactions (Proesmans et al., 2021). The study by Figueroa et al. (2020) found that intensively managed agricultural landscapes in which mass flowering crops are visited for pollination services, could reduce the spread of pathogens (Figueroa et al., 2020). In contrast, other studies found no significant effect of landscape on pathogen prevalence (Meikle et al., 2017; Olgun et al., 2020).

In the same way, there is a differential exposure to agrochemicals depending on the monoculture, the season and the region or country where migratory beekeeping takes place. Several studies have shown the complex interaction between pesticide exposure and the ability of honey bees to resist or tolerate pathogen infection (O'Neil et al., 2018; Harwood and Dolezal, 2020), and how there are country-dependent responses to specific pesticides on honey bees and wild bees (Woodcock et al., 2017). This impact is greater in migratory beekeeping colonies because they are subject to increased pesticide exposure compared to stationary ones which depends on both the crop and the timing of the pollination service (Traynor et al., 2016b). For example, migratory colonies are exposed to a specific combination of several agrochemicals with synergistic toxicity to honey bees in pollination operation in California almond crops (Wade et al., 2019) or high level of pesticide in late cucumber pollination (Traynor et al., 2016b).

Several studies have also highlighted the important role that climatic variables play in disease prevalence and risk in honey bees (Giacobino et al., 2017; Rowland et al., 2021). At a continental scale, however, no relationship has been found between honey bees and viral prevalence, probably due to features of their social life that allow them to regulate hive temperature or maintain food resources in good condition (Piot et al., 2022). However, this study has found a correlation between viral prevalence and climatic variables in wild bees that could impact honey bees by spillback.

There have been just a few studies that have analysed how pathogen prevalence and abundance varied in migratory and stationary hives with the date of sampling or seasonality (i.e. vanEngelsdorp et al., 2013; Alger et al., 2018; Jara et al., 2021; Simone-Finstrom et al., 2022). Some of them identified seasonality as a determining factor for pathogen prevalence (i.e. Runckel et al., 2011; Glenny et al., 2017) even with stronger effects than the type of management (Guimarães-Cestaro et al., 2017; Alger et al., 2018). There are some general trends in pathogen seasonality such an increase of DWV or decrease in *Variomorpha* species as the season progresses. However, this effect varies according to the type of management. For example Simone-Finstrom et al. (2022) showed higher BQCV in the migratory than the stationary colonies early in the season but in late season the opposite trend was found.

Finally, these differences can be explained by the lack of uniformity of the variables analysed, and the methodological heterogeneity across the studies. Small sample sizes, lack of proper control, methodological differences or the timing of sampling increase the heterogeneity of results. For example, the pathogen prevalence changed from the lowest during almond pollination to very high after almond pollination (Cavigli et al., 2016). As a result, the impact of migratory beekeeping on certain pathogens or parasites such as Varroa mites show disparate results with both increasing or decreasing the prevalence. It does not make sense to compare the prevalence of Varroa mites between stationary and migratory hives if there is no information about the number of brood combs or acaricides applied in the colonies as potential significant differences found could be caused by these factors. Therefore, studies aiming to provide conclusive results on the impact of migratory beekeeping on honey bee health should include a comprehensive description of the hives management and should implicitly account for this in their experimental design.

In the same way, most of the studies lack proper controls in their experimental design. Honey bees can travel for long distances looking for floral resources (more than 9.5 km; Beekman and Ratnieks, 2000), and hence, different colonies can exchange pathogens and parasites when sharing floral resources at the landscape level (Graystock et al., 2015). Thus, research analysing the impact of migratory beekeeping on pathogen and parasite prevalence should ensure that their controls (i.e. stationary colonies) are properly isolated from other migratory colonies, otherwise results obtained would be unreliable. In that sense, only two of the studies considered in this review included a control with honey bee hives isolated from the influence of other migratory colonies (Dietemann et al., 2006; Alger et al., 2018) and significant differences between migratory and stationary colonies were only detected in some cases when this factor was considered (Dietemann et al., 2006).

Heterogeneous results obtained in studies assessing the impact of migratory beekeeping on colony losses can be explained by the same factors mentioned above (i.e. biological, geographical, climatic and management factors). Most of the studies that analysed the impact of migratory beekeeping on the genetic change identified an effect on the genetic diversity and structure of honey bee populations. However, there is no empirical evidence about the consequences of these changes on the health of the honey bees and their susceptibility to pests and diseases.

## 5 Conclusion

The information available about the impact of migratory beekeeping on bee health is still scarce. More studies on the issue with robust experimental designs (fine-scale temporal monitoring, pesticides exposures, managements, etc) are urgently required to disentangle the complex interaction between the above-mentioned factors over the pathogen and parasite prevalence on managed and wild bees.

Migratory beekeeping is expected to increase in the future due to climate change (Vercelli et al., 2021) and the increasing demand of managed pollinators to cope with crop pollination. Furthermore, there are no specific studies assessing the role of migratory beekeeping on the spread of pathogens and parasites across wild bee communities which results astonishing given the current worldwide decline of bees. Due to increasing evidence of pathogen transmission from managed to wild bees (Manley et al., 2019; Martinez-López et al., 2021; Nanetti et al., 2021; Piot et al., 2022), there is an urgent need for future studies to assess the impact of migratory beekeeping on wild bees. For this, studies with appropriate experimental designs with a control area free of managed honey bees, and taking into account all the above mentioned factors are needed. A better understanding of transmission dynamics throughout the year is required to detect both spillover and spillback, as well as the role of pathogen transmission between generations of solitary bees via contaminated pollen (i.e. Bramke et al., 2019). Moreover, given the important role of managed honey bees in the transmission of pathogens into wild bee communities, pathogen and parasite management strategies targeting migratory beekeeping could greatly increase the efficiency of controlling the transmission of pathogens in local bee communities. Although the translation of results obtained under controlled conditions to the real world is sometimes complex, a laboratory or semi-field proxy for analysing the effect of transhumant practices is needed to further dissect the detailed mechanisms of pathogen transmission induced by hive movements.

## Funding

VML is funded by a postdoctoral fellowship (21260/PD/19), Fundación Séneca, Región de Murcia (Spain).

## Authors contribution

PDlR, CR and VML conceived and designed the study. CR and VML performed the systematic literature review. CR and VML conducted data generation and curation. PDlR and CR collected the information about migratory beekeeping from official government sites and colleagues. CR, PDlR and VML wrote the first draft of the manuscript. All authors agree with the final version of the manuscript.

## Data availability

The database with all the literature mentioned in this review can be accessed in the Supplemental Table 1.

## Declaration of competing interest

The authors declare that they have no known competing financial interests or personal relationships that could have appeared to influence the work reported in this paper.

The authors of the manuscript entitled “Migratory beekeeping and its influence on the prevalence and dispersal of pathogens to managed and wild bees” declared not conflict of interests.

## References

[bib1] Adgaba N., Al-Ghamdi A., Shenkute A.G., Ismaiel S., Al-Kahtani S., Tadess Y., Abdulaziz M.Q.A. (2014). Socio-economic analysis of beekeeping and determinants of box hive technology adoption in the Kingdom of Saudi Arabia. JAPS.

[bib2] Aizen M.A., Harder L.D. (2009). The global stock of domesticated honey bees is growing slower than agricultural demand for pollination. Curr. Biol..

[bib3] Aizen M.A., Aguiar S., Biesmeijer J.C., Garibaldi L.A., Inouye D.W., Jung C., Seymour C.L. (2019). Global agricultural productivity is threatened by increasing pollinator dependence without a parallel increase in crop diversification. Global Change Biol..

[bib4] Adler L.S., Irwin R.E., McArt S.H., Vannette R.L. (2021). Floral traits affecting the transmission of beneficial and pathogenic pollinator-associated microbes. Curr. Opin. Insect Sci..

[bib5] Alger S.A., Burnham P.A., Lamas Z.S., Brody A.K., Richardson L.L. (2018). Home sick: impacts of migratory beekeeping on honey bee *Apis mellifera* pests, pathogens, and colony size. PeerJ.

[bib6] Allen‐Perkins A., Magrach A., Dainese M., Garibaldi L.A., Kleijn D., Rader R., Montero‐Castaño A. (2021). CropPol: a dynamic, open and global database on crop pollination.

[bib7] Ansari M.J., Al-Ghamdi A., Nuru A., Ahmed A.M., Ayaad T.H., Khan K.A., Al-Waili N. (2017). Diagnosis and molecular detection of *Paenibacillus larvae*, the causative agent of American foulbrood in honey bees in Saudi Arabia. Int. J. Trop. Insect Sci..

[bib8] American Beekeeping Federation (2018). Pollination facts. http://www.abfnet.org/page/PollinatorFacts.

[bib9] Allsopp M.H. (1992). The capensis calamity. S. Afr. Bee J..

[bib10] Bartlett L.J., Boots M., Brosi B.J., de Roode J.C., Delaplane K.S., Hernandez C.A., Wilfert L. (2021). Persistent effects of management history on honeybee colony virus abundances. J. Invertebr. Pathol..

[bib11] Beekman M., Ratnieks F.L.W. (2000). Long‐range foraging by the honey‐bee. *Apis mellifera* L. Funct. Ecol..

[bib12] Bramke K., Müller U., McMahon D.P., Rolff J. (2019). Exposure of larvae of the solitary bee Osmia bicornis to the honey bee pathogen *Nosema ceranae* affects life history. Insects.

[bib13] Brar A.S., Sharma H.K., Rana K. (2018). Colony strength and food reserves of *Apis mellifera* L. under stationary and migratory beekeeping in Himachal Pradesh India. J. Entomol. Zool. Stud..

[bib14] CNCAGR (2011).

[bib15] Calderone N.W. (2012). Insect pollinated crops, insect pollinators and US agriculture: trend analysis of aggregate data for the period 1992 2009. PLoS One.

[bib16] Cavigli I., Daughenbaugh K.F., Martin M., Lerch M., Banner K., Garcia E., Flenniken M.L. (2016). Pathogen prevalence and abundance in honey bee colonies involved in almond pollination. Apidologie.

[bib17] Chen Y.P., Pettis J.S., Collins A., Feldlaufer M.F. (2006). Prevalence and transmission of honeybee viruses. Appl. Environ. Microbiol..

[bib18] Cheptou P.O. (2021). Pollination strategies in the face of pollinator decline. Bot. Lett..

[bib19] Clemson A.A. (1985).

[bib20] Columella L.J.M. (1745). Twelve Books: and His Book Concerning Trees. A. Millar.

[bib21] Colwell M.J., Williams G.R., Evans R.C., Shutler D. (2017). Honey bee–collected pollen in agro–ecosystems reveals diet diversity, diet quality, and pesticide exposure. Ecol. Evol..

[bib22] de Miranda J.R., Meeus I., Yañez O., Piot N., Jara L., Onorati P., Paxton R.J. (2021). Protocols for assessing the distribution of pathogens in individual Hymenopteran pollinators. Researcher Square.

[bib23] Decourtye A., Mader E., Desneux N. (2010). Landscape enhancement of floral resources for honey bees in agro–ecosystems. Apidologie.

[bib24] Drummond F.A., Lund J., Eitzer B. (2021). Honey bee health in Maine wild blueberry production. Insects.

[bib25] Eilers E.J., Kremen C., Smith Greenleaf S., Garber A.K., Klein A.M. (2011). Contribution of pollinator–mediated crops to nutrients in the human food supply. PLoS One.

[bib26] Eischen F.A., Wilson W.T., Pettis J.S., Suarez A., Cardoso–Tamez D., Maki D.L., Rubink W.L. (1990). The spread of *Acarapis woodi* Acari: tarsonemidae in Northeastern Mexico. J. Kans. Entomol. Soc..

[bib27] Faurot-Daniels C., Glenny W., Daughenbaugh K.F., McMenamin A.J., Burkle L.A., Flenniken M.L. (2020). Longitudinal monitoring of honey bee colonies reveals dynamic nature of virus abundance and indicates a negative impact of Lake Sinai virus 2 on colony health. PLoS One.

[bib28] Figueroa L.L., Grab H., Ng W.H., Myers C.R., Graystock P., McFrederick Q.S., McArt S.H. (2020). Landscape simplification shapes pathogen prevalence in plant‐pollinator networks. Ecol. Lett..

[bib29] Garbach K., Morgan G.P. (2017). Grower networks support adoption of innovations in pollination management: the roles of social learning, technical learning, and personal experience. J. Environ. Manag..

[bib30] Giacobino A., Pacini A., Molineri A., Cagnolo N.B., Merke J., Orellano E., Signorini M. (2017). Environment or beekeeping management: what explains better the prevalence of honey bee colonies with high levels of *Varroa destructor*?. Res. Vet. Sci..

[bib31] Glenny W., Cavigli I., Daughenbaugh K.F., Radford R., Kegley S.E., Flenniken M.L. (2017). Honey bee *Apis mellifera* colony health and pathogen composition in migratory beekeeping operations involved in California almond pollination. PLoS One.

[bib33] Gordon R., Bresolin‐Schott N., East I.J. (2014). Nomadic beekeeper movements create the potential for widespread disease in the honeybee industry. Aust. Vet. J..

[bib34] Goulson D., Nicholls E., Botías C., Rotheray E.L. (2015). Bee declines driven by combined stress from parasites, pesticides, and lack of flowers. Science.

[bib35] Grangier V., Belloy L., Charrière J.D., Doherr M.G., Fritsche A., Waldvogel A.S. (2015). Real–time PCR as a decision aid in the control of European foulbrood. J. Apicult. Res..

[bib36] Gray A., Adjlane N., Arab A., Ballis A., Brusbardis V., Charrière J.D., Brodschneider R. (2020). Honey bee colony winter loss rates for 35 countries participating in the COLOSS survey for winter 2018–2019, and the effects of a new queen on the risk of colony winter loss. J. Apicult. Res..

[bib37] Graystock P., Goulson D., Hughes W.O. (2015). Parasites in bloom: flowers aid dispersal and transmission of pollinator parasites within and between bee species. Proc. R. Soc. B: Biol. Sci..

[bib38] Graystock P., Ng W.H., Parks K., Tripodi A.D., Muñiz P.A., Fersch A.A., McArt S.H. (2020). Dominant bee species and floral abundance drive parasite temporal dynamics in plant–pollinator communities. Nat. Ecol. Evol..

[bib39] Guimarães-Cestaro L.G., Alves M.L.T.M.F., Silva M.V.G.B., Teixeira É.W. (2017). Honey bee (*Apis mellifera*) health in stationary and migratory apiaries. Sociobiology.

[bib40] Hallmann C.A., Sorg M., Jongejans E., Siepel H., Hofland N., Schwan H., de Kroon H. (2017). More than 75 percent decline over 27 years in total flying insect biomass in protected areas. PLoS One.

[bib41] Harwood G.P., Dolezal A.G. (2020). Pesticide–virus interactions in honey bees: challenges and opportunities for understanding drivers of bee declines. Viruses.

[bib42] Hung K.L.J., Kingston J.M., Albrecht M., Holway D.A., Kohn J.R. (2018). The worldwide importance of honey bees as pollinators in natural habitats. Proc. R. Soc. B: Biol. Sci..

[bib43] Isaacs R., Williams N., Ellis J., Pitts–Singer T.L., Bommarco R., Vaughan M. (2017). Integrated crop pollination: combining strategies to ensure stable and sustainable yields of pollination–dependent crops. Basic Appl. Ecol..

[bib44] Jara L., Ruiz C., Martín–Hernández R., Muñoz I., Higes M., Serrano J., De la Rúa P. (2021). The effect of migratory beekeeping on the infestation rate of parasites in honey bee *Apis mellifera* colonies and on their genetic variability. Microorganisms.

[bib45] Kishan Tej M., Aruna R., Mishra G., Srinivasan M.R. (2017). Industrial Entomology.

[bib46] Le Conte Y., Ellis M., Ritter W. (2010). Varroa mites and honey bee health: can Varroa explain part of the colony losses?. Apidologie.

[bib47] Lee H., Sumner D.A., Champetier A. (2019). Pollination markets and the coupled futures of almonds and honey bees: simulating impacts of shifts in demands and costs. Am. J. Agric. Econ..

[bib48] Leonhardt S.D., Gallai N., Garibaldi L.A., Kuhlmann M., Klein A.M. (2013). Economic gain, stability of pollination and bee diversity decrease from southern to northern Europe. Basic Appl. Ecol..

[bib49] Manley R., Temperton B., Doyle T., Gates D., Hedges S., Boots M., Wilfert L. (2019). Knock‐on community impacts of a novel vector: spillover of emerging DWV‐B from *Varroa*‐infested honeybees to wild bumblebees. Ecol. Lett..

[bib50] Martínez-López V., Ruiz C., Muñoz I., Ornosa C., Higes M., Martín–Hernández R., De la Rúa P. (2021). Detection of microsporidia in pollinator communities of a mediterranean biodiversity hotspot for wild bees. Microb. Ecol..

[bib51] Meikle W.G., Weiss M., Maes P.W., Fitz W., Snyder L.A., Sheehan T., Anderson K.E. (2017). Internal hive temperature as a means of monitoring honey bee colony health in a migratory beekeeping operation before and during winter. Apidologie.

[bib52] Melicher D., Wilson E.S., Bowsher J.H., Peterson S.S., Yocum G.D., Rinehart J.P. (2019). Long–distance transportation causes temperature stress in the honey bee, *Apis mellifera* Hymenoptera: Apidae. Environ. Entomol..

[bib53] Michener C.D. (2000).

[bib54] Moher D., Liberati A., Tetzlaff J., Altman D.G., Group P. (2009). Preferred reporting items for systematic reviews and meta–analyses: the PRISMA statement. PLoS Med..

[bib55] Nanetti A., Bortolotti L., Cilia G. (2021). Pathogens spillover from honey bees to other arthropods. Pathogens.

[bib56] Oberreiter H., Brodschneider R. (2020). Austrian COLOSS survey of honey bee colony winter losses 2018/19 and analysis of hive management practices. Diversity.

[bib57] Olgun T., Everhart S.E., Anderson T., Wu-Smart J. (2020). Comparative analysis of viruses in four bee species collected from agricultural, urban, and natural landscapes. PLoS One.

[bib58] Ollerton J. (2017). Pollinator diversity: distribution, ecological function, and conservation. Annu. Rev. Ecol. Evol. Syst..

[bib59] Osterman J., Aizen M.A., Biesmeijer J.C., Bosch J., Howlett B.G., Inouye D.W., Paxton R.J. (2021). Global trends in the number and diversity of managed pollinator species. Agric. Ecosyst. Environ..

[bib60] Owen R. (2017). Role of human action in the spread of honey bee (Hymenoptera: Apidae) pathogens. J. Econ. Entomol..

[bib61] Pfeiffer V.W., Crowder D.W. (2022). Factors affecting virus prevalence in honey bees in the Pacific-Northwest, USA. J. Invertebr. Pathol..

[bib62] Pinke G., Dunai É., Czúcz B. (2021). Rise and fall of *Stachys annua* L. L. in the Carpathian Basin: a historical review and prospects for its revival. Genet. Resour. Crop Evol..

[bib63] Piot N., Schweiger O., Meeus I., Yañez O., Straub L., Villamar-Bouza L., de Miranda J.R. (2022). Honey bees and climate explain viral prevalence in wild bee communities on a continental scale. Sci. Rep..

[bib64] Pirk C.W., Human H., Crewe R.M., vanEngelsdorp D. (2014). A survey of managed honey bee colony losses in the Republic of South Africa-2009 to 2011. J. Apicult. Res..

[bib65] Mayhoff K.F.T., Pliny (1906). Digital Version in the Packard Humanities Institute Latin Texts Online.

[bib66] Pocol C.B., Šedík P., Brumă I.S., Amuza A., Chirsanova A. (2021). Organic beekeeping practices in Romania: status and perspectives towards a sustainable development. Agriculture.

[bib67] Poole E.M. (2021). Beekeeping practices and challenges in the Kingdom of Saudi Arabia: adoption of new technologies and selection of bee species. RURALS: Review of Undergraduate Research in Agricultural and Life Sciences.

[bib68] Proesmans W., Albrecht M., Gajda A., Neumann P., Paxton R.J., Pioz M., Vanbergen A.J. (2021). Pathways for novel epidemiology: plant–pollinator–pathogen networks and global change. Trends Ecol. Evol..

[bib69] RIRD (2007).

[bib70] Raven P.H., Wagner D.L. (2021). Agricultural intensification and climate change are rapidly decreasing insect biodiversity. Proc. Natl. Acad. Sci. Unit. States Am..

[bib71] Reilly J.R., Artz D.R., Biddinger D., Bobiwash K., Boyle N.K., Brittain C., Winfree R. (2020). Crop production in the USA is frequently limited by a lack of pollinators. Proc. R. Soc. B: Biol. Sci..

[bib72] Rodríguez-Dehaibes S.R., Otero-Colina G., Villanueva-Jiménez J.A., Corcuera P. (2011). Susceptibility of *Varroa destructor* Gamasida: varroidae to four pesticides used in three Mexican apicultural regions under two different management systems. Int. J. Acarol.

[bib73] Rowland B.W., Rushton S.P., Shirley M.D., Brown M.A., Budge G.E. (2021). Identifying the climatic drivers of honey bee disease in England and Wales. Sci. Rep..

[bib74] Runckel C., Flenniken M.L., Engel J.C., Ruby J.G., Ganem D., Andino R., DeRisi J.L. (2011). Temporal analysis of the honey bee microbiome reveals four novel viruses and seasonal prevalence of known viruses, *Nosema*, and *Crithidia*. PLoS One.

[bib75] Sharma D., Abrol D.P., Ahmad H., Srivastva K., Vir V. (2013).

[bib76] Schäfer M.O., Cardaio I., Cilia G., Cornelissen B., Crailsheim K., Formato G., Neumann P. (2019). How to slow the global spread of small hive beetles, *Aethina tumida*. Biol. Invasions.

[bib77] Simone-Finstrom M., Li-Byarlay H., Huang M.H., Strand M.K., Rueppell O., Tarpy D.R. (2016). Migratory management and environmental conditions affect lifespan and oxidative stress in honey bees. Sci. Rep..

[bib78] Simone-Finstrom M., Strand M.K., Tarpy D.R., Rueppell O. (2022). Impact of honey bee migratory management on pathogen loads and immune gene expression is affected by complex interactions with environment, worker life history, and season. J. Insect Sci..

[bib79] Singh R., Levitt A.L., Rajotte E.G., Holmes E.C., Ostiguy N., vanEngelsdorp D., Cox-Foster D.L. (2010). RNA viruses in hymenopteran pollinators: evidence of inter–taxa virus transmission via pollen and potential impact on non–Apis hymenopteran species. PLoS One.

[bib80] Siviter H., Bailes E.J., Martin C.D., Oliver T.R., Koricheva J., Leadbeater E., Brown M.J. (2021). Agrochemicals interact synergistically to increase bee mortality. Nature.

[bib81] Smith M.R., Singh G.M., Mozaffarian D., Myers S.S. (2015). Effects of decreases of animal pollinators on human nutrition and global health: a modelling analysis. Lancet.

[bib82] Spivak M., Reuter G.S. (2001). *Varroa destructor* infestation in untreated honey bee Hymenoptera: Apidae colonies selected for hygienic behavior. J. Econ. Entomol..

[bib83] Steinhauer N., Kulhanek K., Antúnez K., Human H., Chantawannakul P., Chauzat M.P., vanEngelsdorp D. (2018). Drivers of colony losses. Curr. Opin. Insect Sci..

[bib84] Strauss U., Human H., Gauthier L., Crewe R.M., Dietemann V., Pirk C.W. (2013). Seasonal prevalence of pathogens and parasites in the savannah honeybee Apis mellifera scutellata. J. Invertebr. Pathol..

[bib85] Tokarev Y.S., Huang W.F., Solter L.F., Malysh J.M., Becnel J.J., Vossbrinck C.R. (2020). A formal redefinition of the genera *Nosema* and *Vairimorpha* (Microsporidia: Nosematidae) and reassignment of species based on molecular phylogenetics. J. Invertebr. Pathol..

[bib86] Traynor K.S., Rennich K., Forsgren E., Rose R., Pettis J., Kunkel G., vanEngelsdorp D. (2016). Multiyear survey targeting disease incidence in US honey bees. Apidologie.

[bib87] Traynor K.S., Pettis J.S., Tarpy D.R., Mullin C.A., Frazier J.L., Frazier M., vanEngelsdorp D. (2016). In-hive Pesticide Exposome: assessing risks to migratory honey bees from in-hive pesticide contamination in the Eastern United States. Sci. Rep..

[bib88] USDA (2018).

[bib89] USDA (2021).

[bib90] van Zanten H.H., Mollenhorst H., Klootwijk C.W., van Middelaar C.E., de Boer I.J. (2016). Global food supply: land use efficiency of livestock systems. Int. J. Life Cycle Assess..

[bib92] vanEngelsdorp D., Hayes J., Underwood R.M., Pettis J.S. (2010). A survey of honey bee colony losses in the United States, fall 2008 to spring 2009. J. Apicult. Res..

[bib93] vanEngelsdorp D., Tarpy D.R., Lengerich E.J., Pettis J.S. (2013). Idiopathic brood disease syndrome and queen events as precursors of colony mortality in migratory beekeeping operations in the eastern United States. Prev. Vet. Med..

[bib94] Vercelli M., Novelli S., Ferrazzi P., Lentini G., Ferracini C. (2021). A qualitative analysis of beekeepers' perceptions and farm management Adaptations to the impact of climate change on honey bees. Insects.

[bib95] Wade A., Lin C.H., Kurkul C., Regan E.R., Johnson R.M. (2019). Combined toxicity of insecticides and fungicides applied to California almond orchards to honey bee larvae and adults. Insects.

[bib96] Wardhaugh C.W. (2015). How many species of arthropods visit flowers?. Arthropod–Plant Interact.

[bib97] Woodcock B.A., Bullock J.M., Shore R.F., Heard M.S., Pereira M.G., Redhead J., Pywell R.F. (2017). Country-specific effects of neonicotinoid pesticides on honey bees and wild bees. Science.

[bib98] Zattara E.E., Aizen M.A. (2021). Worldwide occurrence records suggest a global decline in bee species richness. One Earth.

[bib99] Zheng H., Cao L., Huang S., Neumann P., Hu F. (2018). Asian Beekeeping in the 21st Century.

[bib100] Zhu X., Zhou S., Huang Z.Y. (2014). Transportation and pollination service increase abundance and prevalence of *Nosema ceranae* in honey bees (*Apis mellifera*). J. Apic.Res..

